# Genome-wide association mapping for root cone angle in rice

**DOI:** 10.1186/s12284-017-0184-z

**Published:** 2017-10-02

**Authors:** Mathilde Bettembourg, Audrey Dardou, Alain Audebert, Emilie Thomas, Julien Frouin, Emmanuel Guiderdoni, Nourollah Ahmadi, Christophe Perin, Anne Dievart, Brigitte Courtois

**Affiliations:** 1 Cirad, UMR AGAP, F34398 Montpellier Cedex 5, France; 2 Cirad / ISRA-Ceraas, BP 3320 Thies, Senegal; 3 Shanghai Jiao Tong University (SJTU), School of Life Sciences and Biotechnology, Shanghai, 200240 China

**Keywords:** Rice, *Oryza sativa*, *Indica*, *Japonica*, root cone angle, hydroponics, association mapping, GWAS

## Abstract

**Background:**

Plant root systems play a major role in anchoring and in water and nutrient uptake from the soil. The root cone angle is an important parameter of the root system architecture because, combined with root depth, it helps to determine the volume of soil explored by the plant. Two genes, *DRO1* and *SOR1*, and several QTLs for root cone angle have been discovered in the last 5 years.

**Results:**

To find other QTLs linked to root cone angle, a genome-wide association mapping study was conducted on two panels of 162 *indica* and 169 *japonica* rice accessions genotyped with two sets of SNP markers (genotyping-by-sequencing set with approximately 16,000 markers and high-density-rice-array set with approximately 300,000 markers). The root cone angle of all accessions was measured using a screen protractor on images taken after 1 month of plant growth in the Rhizoscope phenotyping system. The distribution of the root cone angle in the *indica* panel was Gaussian, but several accessions of the *japonica* panel (all the bulus from Indonesia and three temperate japonicas from Nepal or India) appeared as outliers with a very wide root cone angle. The data were submitted to association mapping using a mixed model with control of structure and kinship. A total of 15 QTLs for the *indica* panel and 40 QTLs for the *japonica* panel were detected. Genes underlying these QTLs (+/−50 kb from the significant markers) were analyzed. We focused our analysis on auxin-related genes, kinases, and genes involved in root developmental processes and identified 8 particularly interesting genes.

**Conclusions:**

The present study identifies new sources of wide root cone angle in rice, proposes ways to bypass some drawbacks of association mapping to further understand the genetics of the trait and identifies candidate genes deserving further investigation.

**Electronic supplementary material:**

The online version of this article (10.1186/s12284-017-0184-z) contains supplementary material, which is available to authorized users.

## Background

Roots are organs that play several essential roles in plants. Roots are needed to uptake soil water and mineral nutrients and anchor plants to the ground. In rice, several ideotypes of root architecture have been proposed depending on the relative importance given to these different roles. A root system with deep and thick roots and a high root to shoot ratio is needed to enable a better uptake of water, which is found at depth under water stress conditions, (Gowda et al. 2011; Fukai and Cooper 1995). Conversely, for nutrient uptake, ideotypes differing in root depth, number and angle were recommended for immobile nutrients such as phosphorus, which are dominantly located in the cultivated layer of soil, and for nutrients easily soluble in the soil solution and more prone to leaching and diffusion in the profile such as nitrogen (Lynch and Brown, 2001; White et al. 2013). Obviously, a combination of both shallow and deep roots, if physiologically and genetically possible in terms of resource allocation, would be the best option to enable optimal topsoil and subsoil exploration.

Root ideotypes should integrate functional parameters such as water and nutrient use efficiency and plasticity in addition to root architecture, as suggested by Ahmadi et al. (2014). In rice, which can be grown under fully anaerobic conditions (irrigated ecosystems), fully aerobic conditions (upland ecosystems) and conditions alternating between aerobic and anaerobic (rainfed lowland ecosystems), roots play an additional role in oxygen transport to the root tips. Good oxygenation is generally obtained through the development of aerenchyma in the cortex via an apoptosis mechanism but it has been suggested that, in some cases, aerial roots can develop above the soil to provide oxygen to the roots under the soil (Ueno and Sato 1989).

**Fig. 1:**
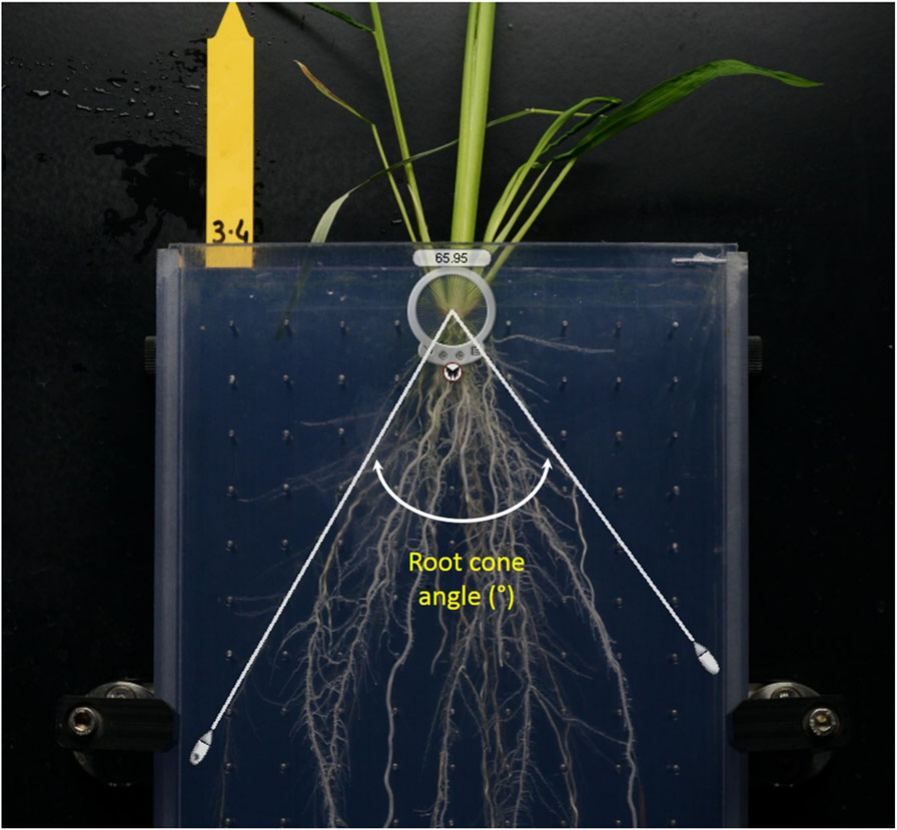
Measurement of the root cone angle with a screen protractor

The spatial distribution of roots in a soil profile is determined by a combination of root growth angle and root length (Abe and Morita 1994). In this paper, the root cone angle designates the angle relative to a vertical axis (Fig. 1). Root cone angle and root depth do not always correlate. In some studies, plants with a narrow root cone angle tended to have increased root depth (Oyanagi et al. (1993) in bread wheat and Kato et al. (2006) in rice) while in other studies, this correlation was absent (Sanguineti et al. (2007) in durum wheat), weak (Uga et al. (2009) in rice), or depended on the soil hydrology and plant growing conditions (Abe and Morita (1994) and Kundur et al. (2015) in rice). While the depth of a root system is particularly complicated to determine, the root cone angle is reasonably easy to measure using specific phenotyping systems such as the basket method (Oyanagi et al. 1993) or the shovelomics method in the field (Trachsel et al. 2011). The broad-sense heritability of root cone angle is moderate to high in cereals so it should be possible to get good responses to selection for this trait (Sanguinetti et al. 2007; Singh et al. 2012). Because the root cone angle could be an important trait to increase crop production under stress conditions (Uga et al. 2015), its natural phenotypic variability and genetic control need to be known.

Large genetic variability has been observed in rice (*Oryza sativa* L.) for different root traits such as root depth, root thickness and root-to-shoot ratio (O’Toole and Bland, 1987). This variability partly follows the genetic organization of the species, which is subdivided into two major components: the *indica* and *japonica* subspecies, which themselves are subdivided into varietal groups and ecotypes within varietal groups (Garris et al. 2005). An ecotype is often characterized by a specific combination of root characteristics that are generally related to its hydrological adaptation (Lafitte et al. 2001). Of course, the ecotype general trends can largely be modified by soil conditions (composition, pH, impedance), soil biology (presence of mycorrhizae or nematodes), water regimes (aerobic or anaerobic or alternating between the two conditions) and genotype x environment interactions. The root cone angle has seldom been considered among the studied architectural root traits, except by Japanese teams who showed that most accessions of some ecotypes (aman, boro and some of the aus varieties within the *indica* subspecies; bulu within the *japonica* subspecies) had a high proportion of crown roots growing horizontally (Ueno and Sato 1992; Kato et al. 2006; Uga et al. 2009). They correlated a wide root cone angle with an absence of response to a gravitropic stimulus (Ueno and Sato 1992).

**Fig. 2:**
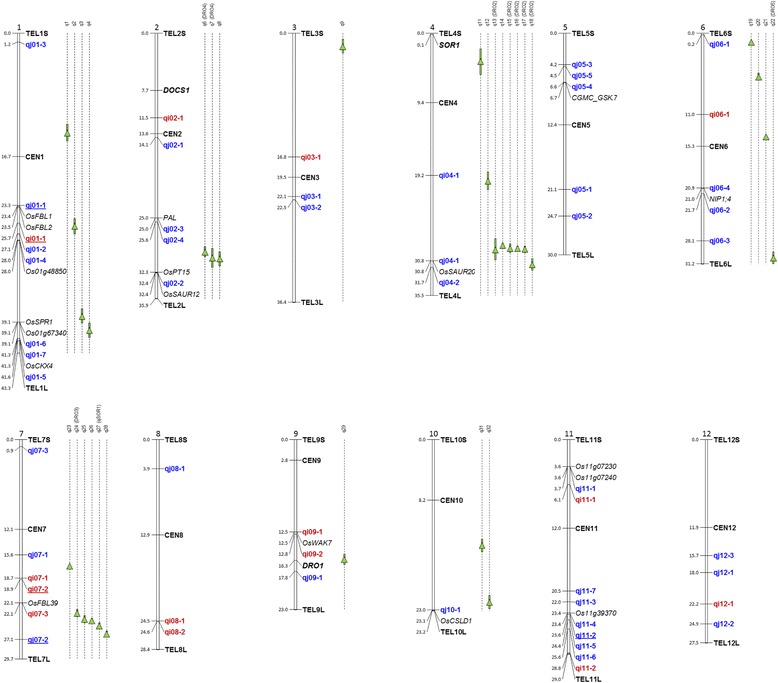
Position of genes and QTLs for root cone angle or related traits In black, genes from the literature listed in Additional file 1: Table S1 and candidate genes from Table 9; in green QTLs from the literature listed in Additional file 1: Table S1; in red (indica panel) and blue (japonica panel), QTLs detected in this study (from Tables 3 to 8); QTLs detected both in GBS and HDRA panels are underlined.

Several teams have studied the genetic control of root cone angle and related traits in rice (Norton and Price 2009; Uga et al. 2012, 2013, 2013, 2015, 2015; Hanzawa et al. 2013; Kitomi et al. 2015; Lou et al. 2015). The detected QTLs and genes are positioned on Fig. 2 and summarized in Additional file 1: Table S1. At least six QTLs named *DRO1* (for *DEEP ROOTING 1*) to *DRO5* were shown to determine the ratio of deep rooting, which is an index based on the frequency of high root growth angles, in a population of approximately 100 recombinant inbred lines (RILs) derived from an indica x japonica cross between IR64 and Kinandang Patong (Uga et al. 2011). Phenotyping was performed using the basket method. Kinandang Patong was the deep-rooted parent with a narrow cone angle. *DRO1*, the major QTL located on chromosome (chr) 9, was cloned (Uga et al. 2011; Uga et al. 2013). The *DRO1* gene (Os09g0439800) was shown to be involved in the root response to gravitropism, downstream of the auxin signaling pathway. The four other QTLs have been fine mapped but not yet cloned (Uga et al. 2013, Uga et al. 2015, Kitomi et al. 2015). Another cross between Gemdjah Beton, a tropical japonica Indonesian variety from the bulu ecotype, and Sasanishiki, a temperate japonica accession from Japan, was used to detect a major QTL named *qSOR1* involvedk in soil surface rooting on chr 7, with other minor QTLs on chr 3, 4 and 6 (Uga et al. 2012). One gene called *SOR1* (for *SOIL-SURFACE ROOTING 1*) was isolated from a mutant population of Nipponbare (Hanzawa et al. 2013). This gene (Os04g0101800) was located on chr 4. A deletion in this gene, which is probably involved in auxin signaling, induced a loss of the gravitropic response in young plants and a soil-surface rooting phenotype.

Several genes or QTLs for root cone angle have been discovered in the last five years, but many remain to be found since the trait appears to be controlled by a combination of genes carrying alleles with major effects, such as the *DRO1* allele from Kinandang Patong, and alleles with smaller effects. Genome-wide association study (GWAS) in natural populations is an efficient tool in rice for localizing QTLs faster and with a much higher resolution than classical genetic approaches using mapping populations (Zhang et al. 2016). GWAS requires a high marker density that is now easily accessible due to GBS (genotyping by sequencing) as described by Elshire et al. (2011) or to the re-sequencing of the rice genome and the discovery of millions of single-nucleotide polymorphisms (SNPs) (Alexandrov et al. 2015). GWAS allows for a precise localization of QTLs due to the rapid decay of linkage disequilibrium (LD) in the association panels. However, rice incurs a high risk of false-positive tests during GWAS because of the bipolar genetic structure of the species. This structural effect can be corrected by using panels specific to each subspecies and by using models that consider subpopulation structure and kinship. Several large panels of rice varieties genotyped with high marker density have been developed for GWAS (e.g., Huang et al. (2010), Zhao et al. (2011), McCouch et al. (2016)). A panel called Orytage composed of 170 *japonica* rice accessions and 204 *indica* rice accessions has been developed by Cirad (Courtois et al. 2013, Lafarge et al. 2017). This panel has been genotyped by GBS and approximately 16,000 markers were detected (SNP and diversity arrays technology (DArT) markers) in the *japonica* panel (Courtois et al. 2013) and a similar number in the *indica* panel (Lafarge et al. 2017). The High-Density Rice Array (HDRA) developed by McCouch et al. (2016) is composed of more than 1500 inbred rice varieties of *Oryza sativa* genotyped with 700,000 SNPs. A very large proportion of the accessions belonging to the Orytage panel are also included in the HDRA panel. The Orytage japonica panel has been used for GWASs on several root traits, including root cone angle (Courtois et al. 2013). However, in this early study, the range of variability in root cone angle was narrowed by trigonometrically deducing this angle from segment measurements and not directly measuring it. Other panels have been used for GWASs for root architectural traits in which root depth was analyzed but not the root cone angle itself (Lou et al. 2015; Phung et al. 2016).

In this study, we used the phenotypic data obtained by directly measuring the root cone angle for sub-samples of the indica and japonica Orytage panels. We conducted GWAS using primarily the GBS data but also the HDRA SNP data. To identify candidate genes associated with the significant markers, we conducted a survey of the genes present in the vicinity of the significant markers with a particular interest given to auxin- and kinases-related genes because genes of these families have already been shown to play a role in determining root cone angle.

## Methods

### Plant materials

A sub-sample of the Orytage panel was used in this study. The *indica* panel was composed of 162 accessions. The *japonica* panel was composed of 169 accessions. The seeds were obtained either from the Centre de Ressources Biologiques Tropicales de Montpellier (http://golo.cirad.fr/FR/) or from the International Rice Research Institute (IRRI) gene bank. The accessions were seed-increased by single-seed-descent over two generations in a Cirad greenhouse. The list of accessions with their country of origin and seed source is given in Additional file 2: Table S2 for the *indica* panel and Additional file 3: Table S3 for the *japonica* panel.

### Genotyping by GBS and filtration of the resulting data

Both panels were genotyped by GBS. The GBS data were available for 156 *indica* and 166 *japonica* accessions. Detailed information on the genotyping procedure is given in Lafarge et al. (2017) for the *indica* panel and Courtois et al. (2013) for the *japonica* panel. Genotyping was conducted at Diversity Arrays Technology Pty Ltd. (DArT P/L), Australia. Briefly, this method involved a step of genome complexity reduction using PstI/TaqI restriction enzymes followed by Illumina short-read sequencing of the restricted products. The resulting sequences, which were trimmed to 69 bp, were aligned to the Nipponbare sequence to determine the position of the restriction sites (DArT markers) and the position of the polymorphisms within the 69-bp sequence (SNP markers). Sequences with no or several positions on the genomes were excluded. Markers with a rate of missing data above 20%, a heterozygosity above 10% or a minor allele frequency (MAF) below 2.5% were discarded. The few remaining heterozygotes (approximately 1.5%) were set as missing data. The missing data were then imputed using Beagle v3.3 (Browning and Browning 2007). The final *indica* and *japonica* sets had 16,232 and 15,921 markers, respectively.

Structure was analyzed on each panel using the program Structure (Pritchard et al. 2000), and the results suggested the existence of 4 subpopulations (I1 to I4) in the *indica* panel (Lafarge et al., 2017) and 6 subpopulations (J1 to J6) in the *japonica* one (Courtois et al. 2013). An accession was assigned to a sub-population when more than 80% of its genome came from that sub-population; otherwise it was classified as admixed (m). For each panel, an unweighted neighbor-joining (NJ) tree based on a dissimilarity matrix computed using a shared allele index was built using DARwin software (Perrier and Jaquemout-Collet 2006) and the sub-population attributions of the accessions were projected as colors on the tree (Additional file 4: Figure S1 and Additional file 5: Figure S2). For the *indica* panel (Additional file 4: Figure S1), I1 (69 acc.) included traditional indica varieties from Asia; I2 (45 acc.) included improved irrigated or upland indica varieties; I3 (16 acc.) corresponded to a special group of indica accessions from Madagascar grown at moderate to high elevation (1250 to 1750 m), and I4 included a small set (5 acc.) of aus or boro accessions from eastern India. The last 21 accessions were classified as admixed. For the *japonica* panel (Additional file 5: Figure S2), J1 included a large set (46 acc.) of improved varieties from Africa, Latin America and Madagascar; J2 (19 acc.) included improved varieties from South-East Asia; J3 included a small set (5 acc.) of improved varieties derived from Colombia 1; J4 (8 acc.) was composed of lowland bulus from Indonesia; J5 (30 acc.) consisted of upland varieties from equatorial Asia, and J6 (10 acc.) comprised temperate accessions from high elevation or high latitudes. The last 48 accessions were classified as admixed. The sub-population attributions are given in Additional file 2: Table S2 and Additional file 3: Table S3.

### Genotyping by HDRA

The HDRA data were recovered from https://ricediversity.org/data/. A subset of accessions corresponding to those used in this study was extracted. The corresponding HDRA ID is given in Additional file 2: Table S2 and Additional file 3: Table S3. For several accessions (35 for the *indica* panel and 20 for the *japonica* panel), HDRA data were not available. The smaller HDRA panel size explains why the GBS data were used as priority and why the two datasets were not merged. The extracted dataset was submitted to the same filtering as were the GBS data, notably to remove markers that became monomorphic within each panel. The missing data were imputed using Beagle v4. The final dataset was composed of 337,150 and 302,942 SNP markers for the *indica* and *japonica* panels, respectively. Few markers were shared with the GBS dataset. All four datasets under HapMap format (indica and japonica GBS and indica and japonica HDRA) are available for download at http://tropgenedb.cirad.fr/tropgene/JSP/interface.jsp?module=RICE under study type “genotypes” as a zip file named “Root cone angle”.

To address discrepancies in names and genotypes between the two genotypic datasets due to differences in seed samples used for the genotyping, a subset of 2000 markers was randomly chosen in each dataset, and two pairs of NJ trees were constructed independently. The GBS and HDRA diversity trees obtained were compared for both subspecies. This procedure enabled the identification of a few accessions that were not classified in the same subspecies or were clearly not the same in the GBS and HDRA panels either because the initial sample was not identical or was heterogeneous or because the sample drifted during the multiplication phase at one site or the other. Since phenotyping was performed with the same seed samples that were used for GBS, these accessions were discarded from the HDRA panels, reducing the size of the HDRA panels to 128 *indica* accessions and 143 *japonica* accessions with both phenotypic and genotypic data. The reduction in panel size did not modify the panel structure, and all subpopulations detected in the GBS panels were present in the HDRA panels.

### Phenotyping

The two panels were phenotyped in the Rhizoscope phenotyping system, which is described in detail by Courtois et al. (2013). Briefly, the plants were grown in 50 × 20 × 2 cm homemade Plexiglas rhizoboxes (internal dimensions) filled with glass beads 5 mm in diameter. Each plate contained a grid of nails that helped maintain the root system in place during all operations. The rhizoboxes were set in tanks (48 rhizoboxes per tank) in which an aerated nutrient solution (modified Hoagland solution; pH 5.4) was circulated. The conditions in the growth chamber were 28 °C during the day and 25 °C at night with a 12:12 photoperiod. The radiation was 400 to 450 μmol photons per m^2^ per s (PAR). The relative humidity was set to 55%. One healthy pre-germinated seed was set on each rhizobox on the top of the beads and allowed to grow. After 28 days of growth, the experiment was stopped. The glass beads were removed from the rhizoboxes, and a high-definition image of the plate with the plant in position was taken. In comparison with the method used by Courtois et al. (2013), the imaging system was set in an aluminum frame enabling all images to be taken from the same distance and with the same magnification. The angle of the root cone to the vertical axis was measured in degrees using a screen protractor (Screen protractor v4.0, iconico.com). This device enabled the rapid measurement of the root cone angle on an image by positioning the center of the see-through visor on the basis of the tillering plateau and aligning the two mobile branches of the protractor to the two most external left and right crown roots (Fig. 1). A few plants that had accidentally been moved from their rhizoboxes during harvest were not measured.

For both panels, the experimental designs were alpha lattices with two replications, and the tank was considered a block effect within replication. The experimental unit was one rhizobox. Since the phenotyping system could only handle 192 plants at once and since 3 × 2 replicated controls (the indica variety IR64 and the japonica variety Azucena) were included in each tank, the replications were grown at two-month intervals.

### Statistics

ANOVA was conducted on the experimental data using a mixed model considering the tank effect to be random. Lsmeans were computed for each accession using SAS v9.2 (SAS Institute, Cary NC, USA). In addition, for each panel, ANOVA was conducted on the accession lsmeans, using sub-populations as the source of variation to assess to what extent the root cone angle was linked to population structure. The admixed accessions were excluded from this specific analysis.

### Association analyses

For all panels, GWAS was conducted with control of kinship among accessions and panel sub-structure using Tassel v5 (Bradbury et al. 2007). As by Courtois et al. (2013), a comparison was first conducted between models with kinship alone and kinship and structure together using an Akaike Information Criterion. The conclusion was that the kinship and structure model best fit the data (data not shown).

For analyses involving the GBS datasets, the percentages of admixture derived from previous analyses (as explained above) were used to control population structure. The kinship matrix was computed using the centered identity-by-state method. Qq (quantile by quantile) plots and Manhattan plots were obtained from Tassel. A threshold of 1e-04 was chosen to declare a marker significant for the GBS datasets. As a comparison, Bonferroni correction would lead to a threshold of approximately 5e-06, and the number of independent tests (Gao 2011) computed using the simpleM program would lead to a threshold of 1e-05 for both panels. To confirm that the chosen threshold was reasonable, the q-values corresponding to the false discovery rates (FDRs) were also computed for each of the significant markers using the formula N*Pmax/n, where N was the total number of markers in the dataset, Pmax was the observed *p*-value and n was the number of markers significant at this threshold (Storey and Tibshirani 2003). Markers that were significant in an interval of less than +/−50 kb were considered to belong to the same QTL.

For analyses involving the HDRA datasets, the number of accessions was 20% and 13% lower than for the GBS data for the *indica* and *japonica* panels, respectively. This difference was important and justified repeating the structure analysis. Principal Component Analyses (PCA) were therefore conducted on the genotypic data; 4 and 6 axes were retained for the *indica* and *japonica* panels, respectively, since the number of sub-populations was unchanged compared to that of the GBS data. A threshold of 5e-06, which considers the fact that the HDRA datasets included approximately 20 times more markers than did the GBS datasets, was chosen to declare a QTL significant. By comparison, Bonferroni correction and the number of independent tests would lead to thresholds of approximately 1e-07 and 5e-07, respectively. The q-values were computed the same way as for the GBS datasets.

In GWAS, a low sample size relative to the number of markers and association tests can lead to inconsistency in the results. To assess the robustness of the detected associations, a sub-sampling procedure was conducted for the GBS data. The method is similar to that used by Tian et al. (2011) on maize except that our association analysis, like that of Lafarge et al. (2017), was based on a single marker model rather than a multiple marker model. A set of 80% of the accessions (125 accessions for the *indica* panel and 133 for the *japonica* panel) was chosen at random without replacement and GWAS was conducted on this set. Random sub-sampling was repeated 100 times. The number of times that an association was detected at *p* < 1e-03 was counted to obtain a sub-sampling posterior probability for each marker. Based on the distribution of the results, a threshold that had a 95% chance of not being overtaken was chosen. Sub-sampling was preferred to bootstrap because of the genetic structure of the panels and the simplicity in file preparation from not having several copies of a given accession. For the HDRA dataset, because of the smaller size of the panel and the much higher number of markers considerably slowing down the analyses, no robustness test was attempted.

### Detection of candidate genes

The position of the significant markers was compared to that of approximately 200 root-related genes in rice from a database established in the framework of the EURoot project (http://euroot.cirad.fr/euroot/JSP/interface.jsp?module=RICE). Then, the function of the candidate genes in which significant markers were detected was determined using OrygenesDB (http://orygenesdb.cirad.fr/). In addition, genes located in intervals of 50 kb on both sides of the marker were explored. This window size was chosen because it was intermediate between the average marker distance (approximately 25 kb in the GBS datasets and 1.5 kb in the HDRA datasets) and the distance at which LD decayed to half its initial level (estimated at 100 kb for the *indica* panel and 150 kb for the *japonica* panel on average across all chromosomes but much more variable in short-range segments). Particular attention was paid to auxin-related genes and to kinases because the cloned genes for root angle belonged to these families. Among kinases, leucine-rich repeat receptor-like kinases (*LRR-RLKs)* were identified using a list of *LRR-RLK* genes in rice and a nested list of *LRR-RLKs* involved in root development established by Dievart et al. (2016) and Fischer et al. (2016).

## Results

**Fig. 3:**
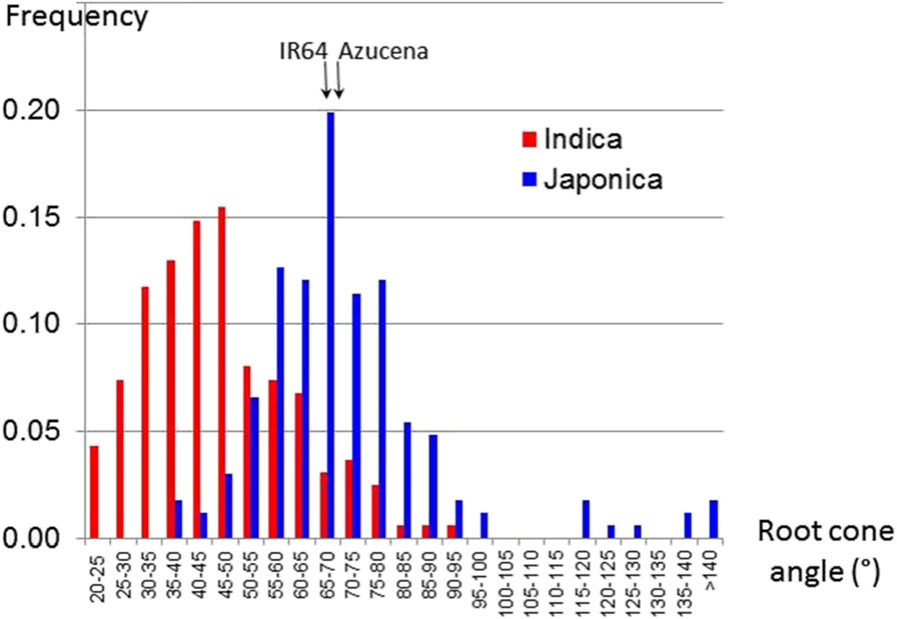
Distribution of the root cone angle (°) for the *indica* (red) and *japonica* (blue) panels

**Fig. 4:**
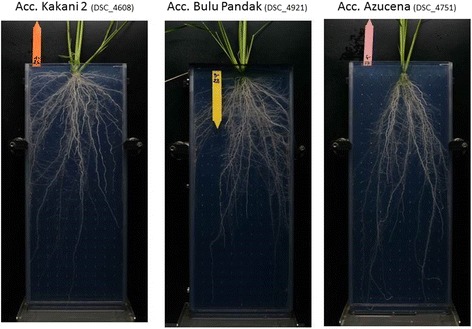
Examples of root cone angle variation within the *japonica* panel. Kakani 2 and Bulu Pandak belong to the bulu ecotype and Azucena (check) is a tropical japonica

Analyses of variance conducted on the root cone angle in different experiments showed that the genotype effect was highly significant for both panels. The mean root cone angle of the two repeated controls (IR64 and Azucena) was similar in the two experiments, with slightly higher values in the experiments involving the *japonica* panel; this similarity enabled a comparison of the distribution of frequency between the two panels (Fig. 3). The *indica* accessions recorded lower values (range from 21.8 to 93.0°) than did the *japonica* accessions (range 36.3 to 164.4°), whose distribution was shifted towards higher values (Table 1). There was discontinuity in the distribution of the *japonica* accessions, with 10 accessions with a very wide root cone angle (above 115°). Examples of extreme accessions are shown in Fig. 4. Seven of these accessions (Bulu Pandak, Cicih Beton, Gundil Kuning, Molok, Padi Boenar, Poenoet Hitam, and Reket Maun) are from Indonesia and encompass all the accessions of subpopulation J4, which corresponds to the bulu ecotype (a special group of rainfed lowland accessions). Three accessions (Gompa2, Jumula 2 and Kakani 2) are from India and Nepal and belong to subpopulation J6, which is composed of temperate japonica accessions. Conversely, the seven other accessions from subpopulation J6 had very acute root cone angles (from 46.9 to 69.9°). When the accessions with very wide root cone angles were removed, the mean of the *japonica* panel dropped to 67.3° (range 36.3 to 96.0°), which was still higher than that of the *indica* panel.

**Table 1:** Statistics of the *indica* and *japonica* panels for the root cone angle

Statistics	*Indica* panel	*Japonica* panel
No. observations	162	166
Minimum	21.8	36.3
Maximum	93.0	164.4
Mean	46.4	71.5
Standard deviation	14.5	19.7
CV of the panel	0.31	0.27
Mean IR64	65.9	68.9
Mean Azucena	67.8	70.1

When undertaking GWAS, a situation in which the phenotypic organization closely parallels the genetic structure is risky since true functional associations can be excluded because these associations are confounded with subpopulation effect. The means of the different subpopulations were computed to assess this risk (Table 2). For the *indica* panel, subpopulation I4 composed of aus/boro accessions was different from the other three subpopulations, showing a narrower root cone angle. For the *japonica* panel, subpopulations J4 (bulus) and J3 (a group of improved upland rice varieties derived from Colombia 1) were significantly different from the other subpopulations, and exhibited contrasting behaviors: J4 with the largest root cone angle and J3 with the narrowest one. Subpopulation J6, in which extremes were pooled, was not significantly different from the three other intermediate subpopulations, probably because of its large variance.

**Table 2:** Mean per subpopulation (admixed excluded)

Sub-population	N	Root cone angle		Subpopulation description
*Indica*				
I1	69	42.6	a	Traditional irrigated varieties
I2	45	52.9	a	Improved varieties (irrigated and upland)
I3	16	50.2	a	Specific group of medium elevation from Madagascar
I4	5	30.0	b	Aus/boro varieties from eastern India
*Japonica*				
J4	8	126.1	a	Rainfed lowland bulus from Indonesia
J6	10	82.2	b	Irrigated temperate rice varieties
J2	19	77.6	b	Upland rice varieties from SE Asia
J5	30	69.9	b	Upland rice varieties from equatorial Asia
J1	46	67.6	b	Upland rice acc. From Africa, Latin America and Madagascar
J3	5	53.2	c	Improved upland rice varieties derived from Colombia 1

### GWAS on the *indica* panel

An initial GWAS analysis was conducted on the *indica* panel using a mixed model with control of structure and kinship (Table 3). This model enabled the control of false positives as seen on the q-q plot (Additional file 6: Figure S3). This analysis enabled the identification of 8 significant markers (*p* < 1e-04), corresponding to 5 different QTLs on chr 3, 6, 8 and 9 (Additional file 7: Figure S4). The q-value of these markers varied between 0.01 and 0.16 (Table 3), which can be deemed reasonable and justifies the chosen threshold. Then, the GWAS was repeated using the same model on 100 subsamples, enabling the computation of a posterior probability (the number of times the marker was considered significant in the 100 subsamples). The distribution of the posterior probabilities showed that no marker had greater than a 5% chance to be detected more than 30 times; therefore, a posterior probability of 0.30 was chosen as a threshold. Based on this criterion, 6 of the 8 original significant markers passed the threshold (posterior probabilities of 0.54 to 0.87). The two markers that did not pass the threshold were also those with the lowest initial probabilities. They were detected only 10 to 17 times. The subsampling also enabled the identification of 6 additional markers corresponding to 5 QTLs that were not detected by the full-size panel initial association analysis. Their original *p*-value, which is indicated in italics in Table 3, varied from 1.69e-03 to 1.02e-04. With the exception of q06–1, the MAF of all 14 of these markers was greater than 5%.

**Table 3:** Markers significant in the GBS *indica* panel

Marker	QTL	Chr	Position	Posterior probability	*p*-value	q- value	Number obs. minor allele
S01_25684060	qi01–1	1	25 684 060	0.42	*1.69E-03*		46
S01_25685951		1	25 685 951	0.41	*1.73E-03*		45
**S03_16707839**	**qi03–1**	**3**	**16 707 839**	***0.17***	**7.12E-05**	**0.14**	**72**
**S03_16748994**		**3**	**16 748 994**	**0.61**	**3.33E-06**	**0.03**	**74**
**S03_16777244**		**3**	**16 777 244**	**0.87**	**3.73E-06**	**0.02**	**61**
**S03_16789846**		**3**	**16 789 846**	**0.66**	**1.90E-05**	**0.08**	**59**
**S06_10959696**	**qi06–1**	**6**	**10 959 696**	**0.75**	**6.81E-05**	**0.16**	**7**
S07_18744029	qi07–1	7	18 744 029	0.45	*6.49E-04*		24
S07_18908531	qi07–2	7	18 908 531	0.53	*9.17E-04*		17
**S08_24524064**	**qi08–1**	**8**	**24 524 064**	**0.54**	**3.25E-05**	**0.11**	**38**
S08_24620154	qi08–2	8	24 620 154	0.51	*1.02E-04*		36
**S09_12474280**	**qi09–1**	**9**	**12 474 280**	**0.83**	**5.65E-07**	**0.01**	**33**
**S09_12772588**	**qi09–2**	**9**	**12 772 588**	***0.10***	**5.85E-05**	**0.16**	**30**
S11_28821256	qi11–1	11	28 821 256	0.59	*1.06E-03*		14

In bold, markers significant in the initial analysis; in italics, initial probability of the markers being significant through sub-sampling

Then, a GWAS was conducted on the HDRA panel (more markers but fewer accessions). The analysis enabled the identification of 9 markers (*p* < 5e-06) corresponding to 7 QTLs (Table 4). Two of these QTLs (q01–1 on chr 1 and q07–2 on chr 2) corresponded to QTLs identified using the GBS data. These two QTLs were also those with the highest MAF. The other significant markers displayed a low MAF (4 to 7%). For these markers with low MAF, because of the very large size differences between the two genotypic classes, the tests based on ANOVA had a greater risk of being biased due to variance heterogeneities and of associations being spurious.

**Table 4:** Markers significant in the HDRA *indica* panel

Marker	QTL	Chr	Position	p-value	q-value	Number obs. minor allele
**S01_25647740**	**qi01–1**	**1**	**25 647 740**	**3.92E-06**		**37**
**S01_25673948**		**1**	**25 673 948**	**2.92E-06**		**37**
S02_11448899	qi02–1	2	11 448 899	1.59E-06		7
S04_19229271	qi04–1	4	19 229 271	1.75E-06		5
**S07_18909031**	**qi07–2**	**7**	**18 909 031**	**1.55E-06**		**14**
S07_22105379	qi07–3	7	22 105 379	2.66E-06		6
S11_06103683	qi11–1	11	6 103 683	1.08E-07		9
S12_22205736	qi12–1	12	22 205 736	1.72E-07		5
S12_22231768		12	22 231 768	9.82E-07		14

In bold, markers or intervals in common with GBS ones

### GWAS on the *japonica* panel

Because of the discontinuity in the distribution of root cone angle within the *japonica* panel, data were first analyzed as the *indica* panel and then subjected to additional analyses. In the first analysis conducted on the whole *japonica* panel (Table 5), the model, although involving both structure and kinship corrections, did not allow for a good control of false positives, as seen in the q-q plot (Additional file 8: Figure S5). Since the distribution was skewed with a long tail in the positive direction, we attempted a log transformation of the data. The transformation improved the normalization of the model residuals and decreased the significance of the associations but identified the same associations.

**Table 5:** Markers significant in the GBS japonica panel

Marker	QTL	Chr	Position1	Position2	Posterior probability	*p*-value	q-value	Number obs. minor allele
**S01_23183181**	qj01–1	**1**	**23 183 181**	**23 351 413**	**0.65**	**3.76E-05**	**0.07**	**6**
**S01_27104121**	qj01–2	**1**	**27 104 121**	**27 146 133**	**0.55**	**8.36E-05**	**0.11**	**8**
S02_14095794	qj02–1	2	14 095 794		0.54	*2.31E-04*		6
S02_32333192	qj02–2	2	32 333 192	32 457 973	0.57	*1.89E-04*		6
**S05_21144094**	**qj05–1**	**5**	**21 144 094**		***0.01***	**3.15E-06**	**0.03**	**5**
**S05_24706117**	**qj05–2**	**5**	**24 706 117**	**24 734 678**	***0.01***	**1.33E-05**	**0.05**	**5**
**S06_00185125**	**qj06–1**	**6**	**185 125**		***0.01***	**4.06E-05**	**0.07**	**8**
**S06_21685315**	**qj06–2**	**6**	**21 685 315**		**0.71**	**3.03E-05**	**0.07**	**14**
**S06_28055840**	**qj06–3**	**6**	**28 055 840**		**0.77**	**2.51E-05**	**0.07**	**13**
**S07_15566174**	**qj07–1**	**7**	**15 566 174**		**0.71**	**8.22E-05**	**0.12**	**6**
**S07_27048508**	**qj07–2**	**7**	**27 048 508**	**27 051 011**	**0.59**	**9.12E-05**	**0.11**	**9**
**S07_27060189**		**7**	**27 060 189**		**0.66**	**4.61E-05**	**0.07**	**8**
**S08_03918947**	**qj08–1**	**8**	**3 918 947**		**0.86**	**2.53E-06**	**0.04**	**12**
S09_17835213	qj09–1	9	17 835 213		0.54	*1.55E-04*		16
**S10_23032257**	**qj10–1**	**10**	**23 032 257**		***0.01***	**9.02E-06**	**0.05**	**12**
S11_03638312	qj11–1	11	3 638 312	3 667 586	0.64	*1.68E-04*		8
S11_23650929	qj11–2	11	23 650 929		0.73	2.41E-05	0.08	5

In bold, marker significant in the intial analysis; in italic initial probability of the markers only significant through sub-sampling. Position1-position 2: limits of intervals including significant markers in complete LD

Among the significant markers, several markers had exactly the same F and *P* values and, upon verification, were exhibiting exactly the same genotypic pattern, which corresponded to a situation of complete LD (r^2^ = 1). These markers were pooled as intervals in Table 5. However, several markers on different chromosomes were also in complete LD: this was the case for 8 markers in an interval between 32.3 and 35.1 Mb on chr 2, for 9 markers in an interval between 3.2 and 6.9 Mb on chr 6 and for 5 markers in an interval between 11.0 and 11.7 Mb on chr 10. This phenomenon can also be seen on the q-q plots and Manhattan plots (straight line at a given probability). These markers with a low MAF discriminated 5 accessions from the rest of the population. These accessions were Gompa 2, Jumula 2 and Kakani 2 (the 3 accessions from Nepal with a wide root cone angle from temperate subpopulation J6), as well as Jumali from Nepal and Lambayque1 from Peru (two admixed accessions with a root cone angle close to the mean of the panel). These last two accessions showed percentages of admixture that did not enable their classification into subpopulation J6; however, for Jumali, this percentage (0.76 in subpopulation J6) was very close to the 0.80 threshold, and for Lambayque1, these percentages (0.60 in subpopulation J6 and 0.33 in subpopulation J4) showed that the accession was intermediate between subpopulation J6 and bulu subpopulation J4. Such markers carried by different chromosomes but in complete LD could not be used for GWAS. Once removed, 13 significant markers corresponding to 12 QTLs remained (Table 5; Additional file 9: Figure S6). Most markers had a low MAF.

Then, as was performed for the indica panel, GWAS was repeated using the same model on 100 subsamples enabling the computation of a posterior probability. In this case as well, the markers that were in complete LD but located on different chromosomes were removed. Based on the distribution of the posterior probabilities, a threshold probability of 0.50 was chosen. Eight of the 12 original significant markers passed the threshold (posterior probabilities of 0.55 to 0.86), and five new significant markers were added (Table 5). By comparison with the indica panel, the significant markers all had a low to very low MAF. We determined which lines had the low-frequency alleles at these significant markers. The lines involved were different from marker to marker and generally included accessions with extremes phenotypes (from subpopulations J4 or J6) associated with one or several accessions with rather average phenotypes from the same or other subpopulations.

A GWAS was then conducted on the japonica panel with the HDRA dataset (Table 6). The same phenomenon was observed as with the GBS data but was more pronounced: long stretches of markers in complete LD on chromosome segments and markers in complete LD located on different chromosomes (up to 828 markers in one case). The analysis enabled the identification of 19 markers (*p* < 5e-06) corresponding to 18 QTLs. Three of these QTLs (qj01–1 on chr 1, qj07–2 on chr 2 and qj11_2 on chr11) corresponded to QTLs identified using the GBS data. As with the GBS data, all the detected QTLs had a very low MAF.

**Table 6:** Markers significant in the HDRA japonica panel

Marker	QTL	Chr	Position 1	Position 2	*p*-value	q-value	Number obs. minor allele
S01_01196103	qj01–3	1	1 196 103		9.68E-07	0.04	14
**S01_22981270**	**qj01–1**	**1**	**22 981 270**	**23 571 718**	**1.29E-06**	**0.04**	**5**
S01_27982591	qj01–4	1	27 982 591		2.30E-06	0.05	8
S01_41631488	qj01–5	1	41 631 488		4.07E-06	0.06	9
S02_24998179	qj02–3	2	24 998 179		3.04E-06	0.07	5
S03_22066093	qj03–1	3	22 066 093		6.27E-10	<0.01	9
S04_30759181	qj04–1	4	30 759 181		1.77E-06	0.04	11
S05_04237081	qj05–3	5	4 237 081	4 240 625	3.17E-06	0.06	5
S05_06615188	qj05–4	5	6 615 188		7.55E-07	0.05	5
S06_20926166	qj06–4	6	20 926 166		1.10E-07	0.02	5
**S07_27039086**	**qj07–2**	**7**	**27 039 086**	**27 067 119**	**5.72E-07**	**0.04**	**6**
S11_21915814	qj11–3	11	21 915 814	21 986 377	1.28E-06	0.04	5
S11_23438923	qj11–4	11	23 438 923		4.00E-06	0.07	6
**S11_23640395**	**qj11–2**	**11**	**23 640 395**		**1.41E-06**	**0.04**	**5**
S11_24364407	qj11–5	11	24 364 407		1.28E-06	0.04	6
S11_24388283		11	24 388 283		9.43E-07	0.05	7
S11_25610599	qj11–6	11	25 610 599		1.43E-07	0.01	8
S12_17977193	qj12–1	12	17 977 193		4.05E-06	0.07	12
S12_24921245	qj12–2	12	24 921 245		3.16E-06	0.06	6

In bold, markers or intervals common with GBS ones

To assess the extent to which these results were influenced by the group of 10 accessions with a very wide root cone angle, we repeated the same analyses on the panel excluding those 10. This time, the model considering kinship and structure accurately controlled the number of false positives (Additional file 10: Figure S7). No marker was significant at *p* < 1–04, probably because of the drastic reduction in the range of phenotypic values. The sub-sampling, with a threshold of 0.16 this time, identified six markers and five QTLs that all corresponded to the markers significant at p < 5-e04 in the initial analyses (Table 7; Additional file 11: Figure S8). None of these markers had a low or very low MAF. These results showed that the exclusion of the 10 outlier accessions allowed the targeting of another set of genes involved in root cone angles. A GWAS was then conducted on the reduced *japonica* panel with the HDRA dataset (Table 8). Four markers on chr 2, 3, 7, and 12 were significant but had a relatively high qvalue (from 0.17 to 0.36). None of these markers were shared with those detected in the GBS dataset.

**Table 7:** Markers significant in the GBS *japonica* panel excluding the 10 extreme lines with very wide root cone angles

Marker	QTL	Chr	Position1	Position2	Posterior probability	*p*-value	Number obs. minor allele
S01_39102231	qj01–6	1	39 102 231		0.19	*4.75E-04*	34
S01_41207248	qj01–7	1	41 207 248	41 304 330	0.30	*2.08E-04*	17
S01_41249221		1	41 249 221		0.40	*1.42E-04*	18
S04_31661682	qj04–2	4	31 661 682		0.21	*4.56E-04*	62
S05_04482655	qj05–5	5	4 482 655		0.31	*2.88E-04*	29
S11_20481199	qj11–7	11	20 481 199		0.16	*4.26E-04*	61

In italics, initial probability of the markers only significant through sub-sampling. Position 1-position 2: limits of intervals including significant markers in complete LD

**Table 8:** Markers significant in the HDRA *japonica* panel excluding the 8 extreme lines with very wide root cone angle

Marker	QTL	Chr	Position	*p*-value	q-value	Number obs. minor allele
S02_25551591	qj02–4	2	25 551 591	5.71E-07	0.17	9
S03_22524470	qj03–2	3	22 524 470	4.76E-06	0.36	5
S07_00929748	qj07–3	7	929 748	2.81E-06	0.28	5
S12_15655309	qj12–3	12	15 655 309	2.40E-06	0.36	21

To identify the loci responsible for the very wide root cone angle, assuming that this trait can be controlled by a major gene, we tested other approaches using only the GBS dataset since phenotypic and genotypic data were available for 10 of the extreme accessions compared with only 8 in the HDRA dataset. First, we looked at markers that were discriminant between the 10 accessions with an open root cone angle and the rest of the panel (157 accessions). Marker S06_21,685,315, corresponding to qj06_02 was found to be the one that discriminated the best the two phenotypic groups. The variant allele present in the 10 accessions with a wide root cone angle was present in only four accessions of the rest of the panel. However, these four accessions included Ketan Lumbu (bulu) and Padi Kasale (close to bulus), which had wide root cone angles (close to 100°), as well as NPE253 and NPE826 (temperate japonica) from Pakistan, which had narrow angles (approximately 50°). In contrast, when the reference allele was present in the 10 accessions, no marker was found discriminant with at best a ratio of 7 accessions with the reference allele compared with 150 accessions with the variant allele in the rest of the panel.

These results led us to assume that the wide root cone angle trait could be controlled by different mutations in the J4 and J6 subpopulations. It was not possible to test this hypothesis with J4 since all alleles specific to the subpopulation would have appeared associated with the mutation. However, it was possible to avoid structural problems with J6, since J6 was composed of only accessions of the two tails of the distribution. The same approach as above was therefore applied to J6. The allele frequency among the three accessions of J6 with open root angles was compared to the allele frequency among the 7 accessions with narrow root angles. Because of the very small size of the sample, only the extreme situation (one allele present in one group and absent in the other or vice versa) was considered. This time, 367 and 132 markers distributed in cluster on different chromosomes were found discriminant within J6 for the variant and the reference alleles, respectively. However, all these markers segregated in the rest of the population, with at best a ratio of 2 accessions with the reference or variant alleles compared with 155 with the complementary one.

We ended with 15 QTLs for the *indica* panel and 40 QTLs for the *japonica* panel (Fig. 2). None of the significant markers were located in similar zones in the *indica* and *japonica* panels.

### Analysis of the candidate genes

The positions of these 55 QTLs were compared to the position of root-related genes in rice from the EURoot database. No known root gene corresponded to the markers that were found to be significant in the *indica* panel. For the *japonica* panel, five genes present in the EURoot database co-localized with significant markers: *SHORT POSTEMBRYONIC ROOTS 1* (*OsSPR1* / Os01g67290) 43.3 kb from qj01_6, *CYTOKININ OXIDASE/DEHYDROGENASE 4* (*OsCKX4* / Os01g71310) within qj01_7, *PHOSPHATE TRANSPORTER 15* (*OsPT15* / Os02g52860) 9.9 kb from qj02_2, *NOD26-LIKE INTRINSIC P 1;4* (*OsNIP1;4* / Os06g35930) 34. 5 kb from qj06–4 and *CELLULOSE SYNTHASE-LIKE FAMILY D* (*OsCSLD1* / Os10g42750) 30.2 kb from qj10_1.

Finally, the genes included in an interval of +/− 50 kb around the markers were analyzed. We found 489 genes with an annotated function, among which 92 were localized within a QTL, and 91 were located in a 10-kb zone around a significant marker (Additional file 12: Table S4). Among these genes, based on Michigan State University annotations (http://rice.plantbiology.msu.edu/), 29 genes were kinases, including 6 *LRR-RLKs*, and 5 genes were linked to the auxin pathway (three 3F–box domain and LRR- containing proteins and two members of the auxin-responsive *SAUR* gene family). The 17 genes for which a link with roots could be found either in rice or through orthology with *Arabidopsis* are summarized in Table 9.

**Table 9:** List of the most interesting genes underlying the QTLs

Gene	Chr	Start position	Stop position	QTL	Distance from mk (in kb)	Kinase	Kinase sub-group	Annotation (MSU)
Os01g41340	1	23 392 955	23 394 611	qj01–1	IN	No		*OsFBL1*; F-box domain- and LRR-containing protein
Os01g41530	1	23 510 098	23 512 343	qj01–1	IN	No		*OsFBL2*; F-box domain- and LRR-containing protein
Os01g48850	1	28 028 738	28 032 031	qj01–4	46.1	No		Auxin-responsive protein putative
Os01g67290	1	39 052 262	39 058 974	qj01–6	43.3	No		*OsSPR1*; cyclin-related protein putative
Os01g67340	1	39 091 728	39 095 339	qj01–6	6.9	Yes	RLCK-VIII	*STRUBBELIG-RECEPTOR FAMILY 8* precursor putative
Os01g71310	1	41 300 203	41 303 044	qj01–7	IN	No		*OsCKX4*; cytokinin dehydrogenase precursor
Os02g41650	2	24 985 255	24 989 388	qj02–3	8.8	No		Phenylalanine ammonia-lyase protein (PAL) putative
Os02g52860	2	32 319 364	32 323 332	qj02–2	9.9	No		*OsPT15*; phosphate carrier protein mitochondrial precursor
Os02g52990	2	32 418 493	32 419 361	qj02_2	IN	No		*OsSAUR12*; auxin-responsive SAUR gene family member
Os04g51890	4	30 783 636	30 784 530	qj04–1	24.4	No		*OsSAUR20*; auxin-responsive SAUR gene family member
Os05g11730	5	6 657 481	6 661 493	qj05–4	42.3	Yes	GSK2	*CGMC_GSK.7*; CGMC includes CDA, MAPK, GSK3, and CLKC kinases
Os06g35930	6	20 960 706	20 961 819	qj06–4	34.5	No		*NIP1;4*; aquaporin protein, putative
Os07g36900	7	22 095 130	22 099 277	qi07–3	6.1	No		*OsFBL39*; F-box domain and LRR-containing protein
Os09g20740	9	12 494 569	12 498 572	qi09–1	20.3	Yes	WAK	*OsWAK79*; OsWAK receptor-like protein kinase
Os10g42750	10	23 062 454	23 066 292	qj10–1	30.2	No		*OsCSLD1*; cellulose synthase-like family D
Os11g07230	11	3 633 303	3 638 663	qj11–1	IN	Yes	LRR-XII	Receptor kinase, putative
Os11g07240	11	3 640 868	3 644 003	qj11–1	IN	LRR-RLK	SG_XIIa-6c	Serine/threonine-protein kinase BRI1-like 2 precursor, putative
Os11g39370	11	23 431 233	23 436 807	qj11–4	2.1	Yes	*OsSERL1*	*BRASSINOSTEROID INSENSITIVE 1*-associated receptor kinase 1 precursor

## Discussion

We ran GWASs on root cone angle in *indica* and *japonica* panels of intermediate size, which were genotyped with two large sets of markers. The root cone angle was assessed in a total population of 331 accessions and enabled the identification of accessions with exceptionally wide root cone angles. This phenotype seems relatively frequent in *O. sativa* and distributed in different varietal groups. In our *japonica* panel, these accessions included all those belonging to the bulu ecotype, confirming the results obtained by Ueno and Sato (1989, 1992) and a few temperate japonica lines from South Asia that had not yet been identified as carrying such mutations. Ueno and Sato (1992) also found accessions with wide root cone angle among the aus and boro ecotypes from India and tjereh from Indonesia that belong to the *indica* subspecies. We did not find any such line in our *indica* panel, but these specific ecotypes were represented by very few accessions, probably too few to detect the mutation. Not considering mutants, the root cone angles of the indica and japonica panels largely overlapped, while indica accessions are said to be generally shallow rooted and japonica accessions to be deep rooted (Lafitte et al. 2001). One could wonder whether this result is specific to our phenotyping system. However, Uga et al. (2009), using the 3D basket system, also concluded that there was no significant difference in the distribution of an index based on root cone angle between the indica and japonica groups. On an accession basis, Clark et al. (2011), using a gellan gum culture system, did not find differences in root initiation angle between IR64 and Azucena, our two controls in the present study that are nonetheless known to have contrasting root depths (Yadav et al. 1997). These results confirm those from Abe and Morita (1994) that the link between narrow root cone angle and increased root depth cannot be generalized in rice.

Since our panels had been genotyped by two different methods (GBS and high-density chip), we assessed whether the number of markers was a limitation for association detection by comparing the results obtained with the GBS data sets (approximately 15,000 markers) and the HDRA datasets (approximately 300,000 markers). Theoretically, the large increase in marker density should not lead to the identification of many more associations because, in both the *indica* and the *japonica* panels, LD decay is much slower than the average distance between markers even in the two GBS datasets (Courtois et al. 2013; Lafarge et al. 2017). However, this statement is based on averages, and locally, large variations or ruptures in LD pattern can be observed, because of recent mutations in some lineages, for example. In such cases, the HDRA dataset has a greater chance of showing a higher LD between the causative polymorphism and the genotyped markers than does the GBS dataset. Based on our data, the HDRA dataset associations recorded much lower *p*-values than did those from the GBS dataset (minimum *p*-value of 6.3e-10 for the HDRA compared with 2.5 e-06 for the GBS), but when using a threshold that considered the difference in the number of markers between datasets, little difference in the number of significant associations was observed due to the 20-fold-increase in marker density between the GBS and HDRA datasets. However, one cannot exclude that this lack of difference results partly from the panel sizes, which are smaller for the HDRA datasets. Improvement in resolution can be obtained by an increase in panel size. In our case, this may have been achieved by pooling the *indica* and *japonica* panels. We did not attempt to do so since the conditions for pooling the datasets were not optimal. The experiments with the two panels were conducted independently, and the common controls (IR64 and Azucena) that could have enabled us to bridge the phenotypic datasets registered similar values for root cone angle, making them poor controls for this trait although showing major differences in other root traits.

For both panels, the significant markers in the GBS and HDRA datasets pinpointed to similar chromosomal segments in several cases but there were also chromosomal segments where the markers were significant for one dataset and not for the other. One possible reason, besides the difference in panel sizes mentioned above, is the fact that the two datasets were submitted to different ascertainment biases in SNP selection. The HDRA dataset, derived from sequence data, is enriched in SNPs in and around genes (McCouch et al. 2016) while the markers of the GBS dataset were more randomly selected. The proportion of markers with a low MAF of the HDRA dataset was also much higher than that of the GBS dataset (63% and 69% of markers with MAF < 10% for the indica and japonica HDRA panels compared with 38.0 and 46.2% for the indica and japonica GBS panels, respectively) although the same filtering procedure was used. Better balanced genotypic classes, as in the GBS dataset, generally provide higher power for association detection.

We detected significant associations and tested the robustness of those associations using a resampling technique. Resampling enables the evaluation of the sensitivity of the tests to the specificities of the samples (Tian et al. 2011). Some of the QTLs were much better supported than others. While we did not encounter methodological difficulties in conducting GWAS in the *indica* panel, we did in the *japo*nica panel. The root cone angle in the *japonica* panel is a clear example of a trait for which GWAS may not be the best way to quickly identify the underlying gene(s). Two problems surface. The first problem comes from the fact that one subpopulation, J4, consisting of accessions from the bulu ecotype, is composed of only one-tail extremes. In such case, correcting population structure will remove the significant associations. The second problem is that, in subpopulation J6, the number of extreme accessions is very low (only 3 accessions) and GWAS is not appropriate for analyzing situations with rare alleles (Gibson 2012; Zhang et al. 2016). In addition, as noted by Korte and Farlow (2013), rare alleles that are specific to such small sub-groups of individuals will be in complete LD, which is what we observed with markers with the same *p*-value on different chromosomes and drove us to eliminate many significant markers. Reverting to classical mapping populations such as RILs deriving from two divergent parents will solve these problems of the confounding effect of population structure and low frequency alleles. The poorer resolution, which is generally registered in classical mapping populations in comparison with GWAS panels, can easily be resolved by increasing the population size. Such a population was developed by Uga et al. (2012) who crossed a temperate japonica with a narrow root cone angle with a bulu with an open root cone angle. A mapping population involving one extreme parent of subpopulation J4 and one from J6 is another option if the objective is to determine whether the phenotypes observed in the various sub-populations are allelic.

Several root-related genes were identified under or close to the significant markers. We screened the literature to determine the biological function of these genes in rice or that of their orthologs in Arabidopsis when no other information was available. First, five genes were found in the EURoot database. Three of them were described as involved in root nutrient transport, in water transport and in root hairs. *OsPT15* (Os02g52860) codes for a mitochondrial phosphate transporter (Liu et al. 2011). *OsNIP1;4* (Os06g35930) is an aquaporin that is expressed in leaf blades and in roots involved in water transport in rice (Sakurai et al. 2008). *OsCSLD1* (Os10g42750) is implicated in the growth of the primary cell wall at the root hair tip. The two other genes are described as affecting root architecture. *OsSPR1* codes for a mitochondrial protein involved in the maintenance of Fe homeostasis. An *OsSPR1* mutant has deficient root elongation in primary, adventitious and lateral roots due to the reduction in root cell length (Jia et al. 2011). *OsCKX4*, which codes for an enzyme responsible for cytokinin degradation, positively regulates crown root initiation and development in rice (Gao et al. 2014). This gene possesses an auxin response element in its promoter, which is the target of the auxin-response factor *OsARF25*. Auxin induces *OsCKX4* gene expression in roots. Knock-out lines of *OsCKX4* show an inhibition of the primary roots and fewer crown roots at the seedling stage. Conversely, lines over-expressing *OsCKX4* show enhanced root system development.

We also identified five genes related to the auxin signaling pathway and auxin response. Three of these genes belong to the F-box-domain- and LRR-containing protein family, but no biological function has been assigned to the three specific FBLs found in this study. The FBL family, which is a subdivision of the large F-Box family, includes only 61 members (Jain et al. 2007). Only a few of them (*OsFLB16* (*AFB2*), *OsFLB21* (*TIR1*) and *DWARK3* (*OsFLB27*)) have been biologically studied. All were shown to have an impact on root architecture, notably on crown and lateral root development. *OsFBL16* and *OsFBL21* interact with *OsIAA1* and alter auxin signaling (Bian et al. 2012). *OsSAUR12* and *OsSAUR20* belong to an auxin responsive family of 58 genes in rice. They both encode a protein bearing an auxin-inducible domain of 118 AA, but its biological function has not yet been determined in rice. However, *OsSAUR1*2 is down-regulated in the mutant of the *CROWN ROOTLESS 1* (*CRL1*) gene (Coudert et al. 2011). *CRL1* is involved in crown root development and is induced by auxin (Inukai et al. 2005). The last auxin-related gene identified, Os01g48850, encodes a putative auxin-responsive protein that has not been studied in rice, but its ortholog in Arabidopsis, At3g61750, is member of the family of DOMON-containing proteins, of which some members are involved in root development (Preger et al. 2009).

Several kinases have an effect on root gravitropism responses (reviewed by Armengot et al. (2016)). In our study, we found 29 kinases belonging to different subfamilies. We identified six *LRR-RLKs*, two *L-LECTIN* kinases, three *RECEPTOR-LIKE CYTOPLASMIC KINASES* (*RLCKs*), one *WAK*, and a few other kinases belonging to different subgroups (Additional file 12 Table S4), but no biological function had been assigned to most of these genes in either rice or Arabidopsis. Among the *LRR-RLKs* with biological function, Os11g07240 (SG_XIIa), which belongs to a cluster of 5 *LRR-RLK* genes on chr11, has been studied by Chen et al. (2013). Os11g07240, called *HYBRID WEAKNESS 1* (*Hw1*), is proposed to be responsible for autoimmunity activation leading to a hybrid weakness phenotype. A near-isogenic line (NIL) carrying an *O. rufipogon* allele at this gene in an elite variety background displays defaults in root development and notably fewer crown roots due to a drastic diminution of crown root primordia. Os11g39370 encodes a protein annotated *BRASSINOSTEROID INSENSITIVE 1*-associated receptor kinase 1 precursor and belongs to the *SERK* family (LRR-RLK SG_II). It is classified in the same SG as *DOCS1*, which is known to affect root cone angle (Bettembourg 2016), but to date, no function has been assigned to this gene. Among the other *LRR-RLKs*, Os01g67340 codes for a protein annotated *STRUBBELIG-RECEPTOR FAMILY* protein that has not yet been studied in rice. In Arabidopsis, mutants of a member of the same family, *SCRAMBLED* (*SCM*), are affected in root hair specification (Kwak and Schiefelbein 2007).

Lastly, Os02g41650, which is part of a cluster of three genes with similar biochemical function, codes for a putative phenylalanine ammonia-lyase (PAL) protein and has recently been studied in relation to gravitropism (Hu et al. 2013). It was hypothesized that this gene contributed to the synthesis of kaempferol and myricetin (two flavonols) in shoot tissues and regulated the transport of auxin during gravitropism.

## Conclusions

The root cone angle is an important trait because it largely determines the volume of soil that a plant explores. In the present study, we identified new sources of large root cone angle in a temperate japonica subpopulation in addition to those already known from the bulu ecotype. We encountered difficulties in using GWAS for this trait in *japonica* rice and proposed other approaches for further genetic analyses. From candidate gene analysis, we identified eight genes close to significant markers (*OsSAUR12* in qj02–2, *OsCKX4* in qj01–7, *OsSPR1* near qj01–6, *OsCSLD1* near qj10–1, *STRUBBELIG-RECEPTOR FAMILY 8* near qj01–6, *BRASSINOTEROID INSENSITIVE 1* near qj11–4, *Hw1* near qj11–1 and *PAL* near qj02–3) that seem to be candidates deserving further investigation.

## Additional files

Additional file 1: Table S1. List of QTLs and genes for root growth angle and related traits (XLSX 16 kb)

Additional file 2: Table S2. List of the accessions included the indica panel with their phenotype. (XLSX 25 kb)

Additional file 3: Table S3. List of the accessions included the japonica panel with their phenotype. (XLSX 27 kb)

Additional file 4: Figure S1. Neighbor joining tree of the *indica* panel. The colors corresponds to the subpopulations defined by Structure. The accessions in black are admixed (PPTX 163 kb)

Additional file 5: Figure S2. Neighbor joining tree of the *japonica* panel. The colors corresponds to the subpopulations defined by Structure. The accessions in black are admixed (PPTX 158 kb)

Additional file 6: Figure S3. Q-q plot for root cone angle in the indica panel with GBS data. (JPEG 68 kb)

Additional file 7: Figure S4. Manhattan plot for the indica panel with GBS data. *P*-values by chromosome for root cone angle. (JPEG 98 kb)

Additional file 8: Figure S5. Q-q plot for root cone angle in the japonica panel with GBS data. (JPEG 73 kb)

Additional file 9: Figure S6. Manhattan plot for the japonica panel with GBS data. *P*-values by chromosome for root cone angle. (JPEG 142 kb)

Additional file 10: Figure S7. Q-q plot for root cone angle for the japonica panel, 10 extreme lines excluded, with GBS data. (JPEG 67 kb)

Additional file 11: Figure S8. Manhattan plot for the japonica panel, 10 extreme lines excluded, with GBS data. *P*-values by chromosome for root cone angle. (JPEG 131 kb)

Additional file 12: Table S4. List of genes with annotated function underlying the QTLs (XLSX 47 kb)
