# Supplementary material for: Genome-wide association mapping for root cone angle in rice
Source: Rice (N Y). 2017 Oct 2;10:45. doi: 10.1186/s12284-017-0184-z (PMC5624858; doi:10.1186/s12284-017-0184-z)
Supplement: Supplementary file 4 — Neighbor joining tree of the indica panel. The colors corresponds to the subpopulations defined by Structure. The accessions in black are admixed (PPTX 163 kb) [file 12284_2017_184_MOESM4_ESM.pptx]

## Slide 1
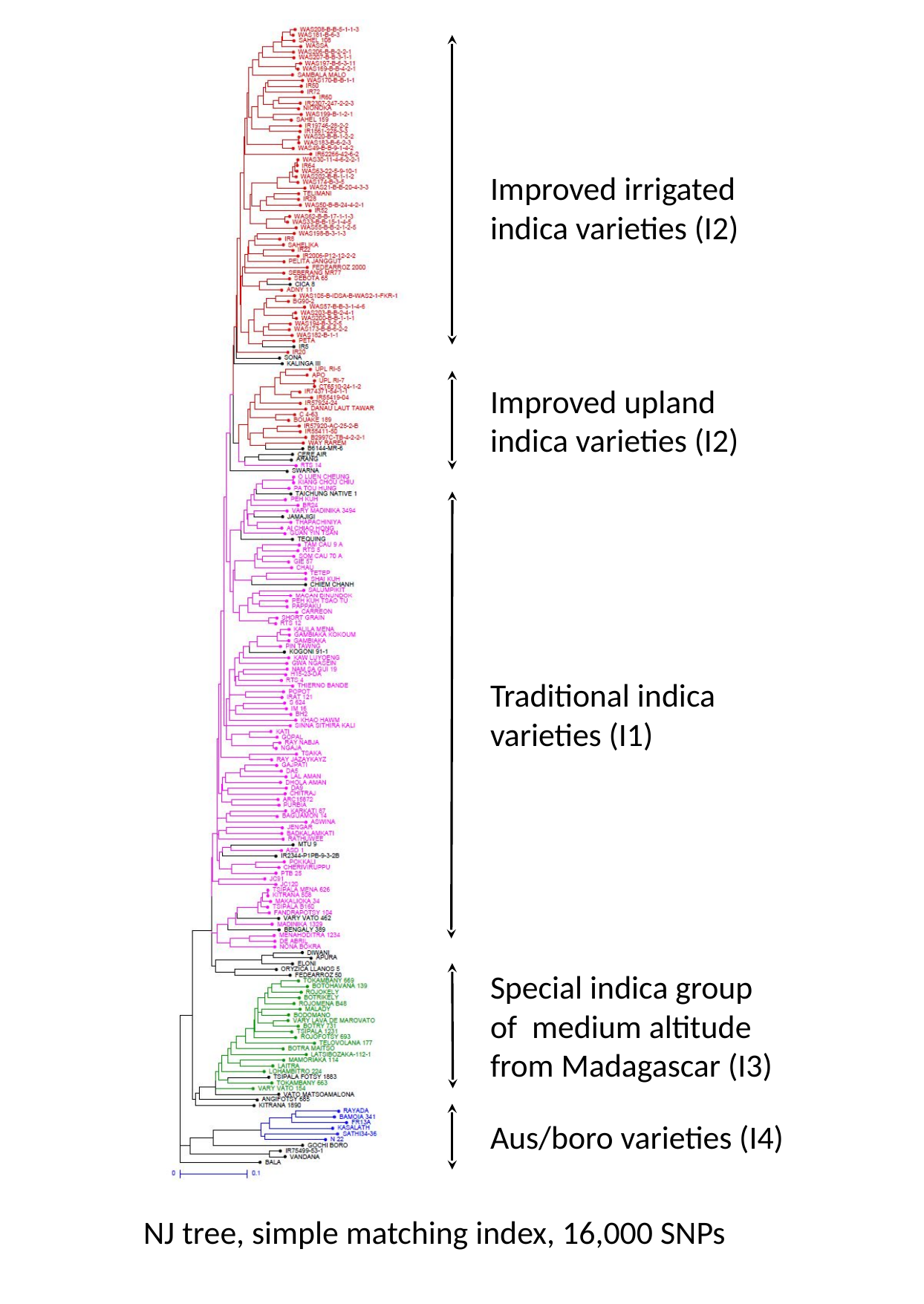

Improved irrigated indica varieties (I2)
Improved upland indica varieties (I2)
Traditional indica varieties (I1)
Special indica group of medium altitude from Madagascar (I3)
Aus/boro varieties (I4)
NJ tree, simple matching index, 16,000 SNPs
