# Supplementary material for: Genome-wide association mapping for root cone angle in rice
Source: Rice (N Y). 2017 Oct 2;10:45. doi: 10.1186/s12284-017-0184-z (PMC5624858; doi:10.1186/s12284-017-0184-z)
Supplement: Supplementary file 5 — Neighbor joining tree of the japonica panel. The colors corresponds to the subpopulations defined by Structure. The accessions in black are admixed (PPTX 158 kb) [file 12284_2017_184_MOESM5_ESM.pptx]

## Slide 1
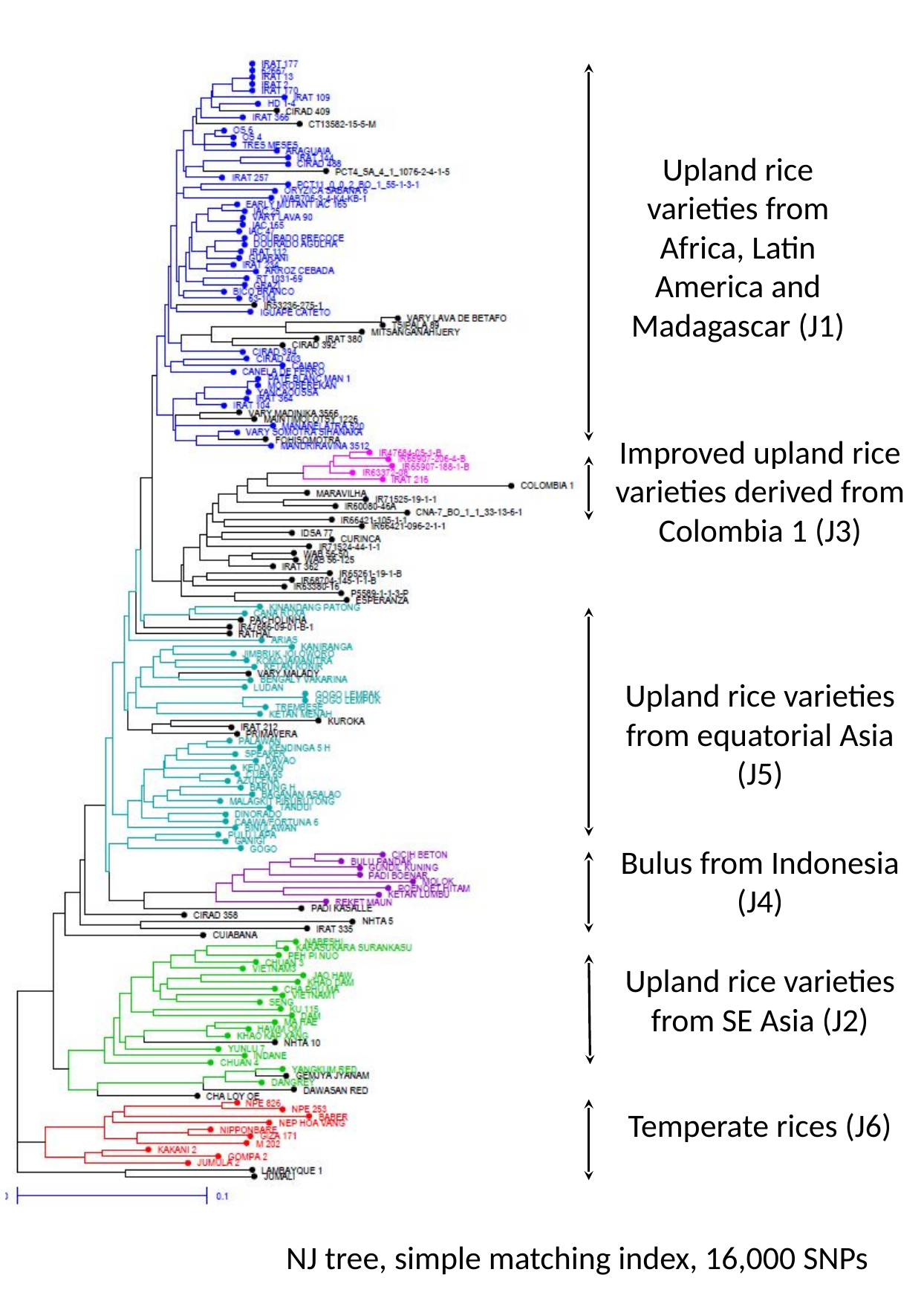

Upland rice varieties from Africa, Latin America and Madagascar (J1)
Improved upland rice varieties derived from Colombia 1 (J3)
Upland rice varieties from equatorial Asia (J5)
Bulus from Indonesia (J4)
Upland rice varieties from SE Asia (J2)
Temperate rices (J6)
NJ tree, simple matching index, 16,000 SNPs
