# Supplementary figures and images for: Genome-wide association mapping for root cone angle in rice
Source: Rice (N Y). 2017 Oct 2;10:45. doi: 10.1186/s12284-017-0184-z (PMC5624858; doi:10.1186/s12284-017-0184-z)

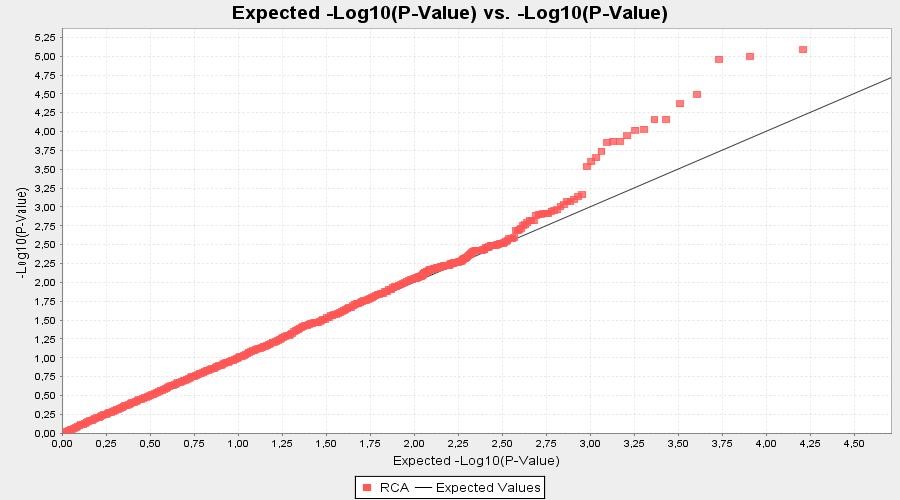

Supplement: Supplementary file 6 — Q-q plot for root cone angle in the indica panel with GBS data. (JPEG 68 kb) [file 12284_2017_184_MOESM6_ESM.jpg]

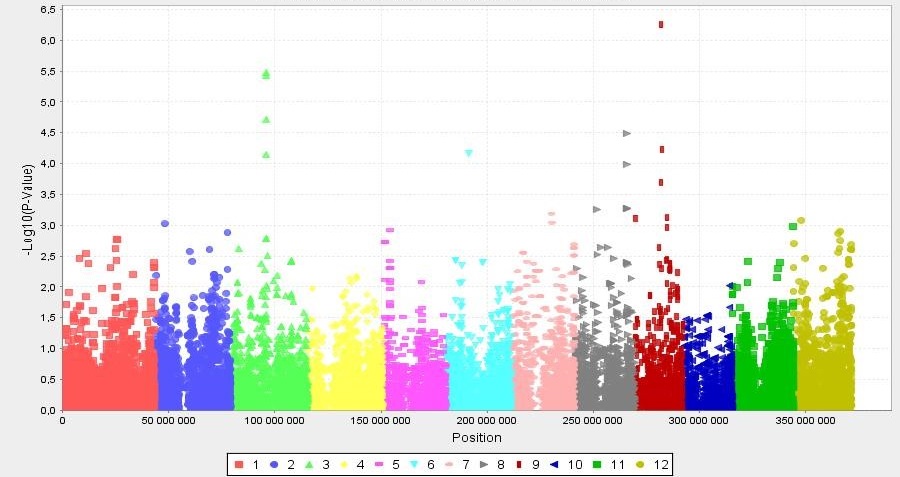

Supplement: Supplementary file 7 — Manhattan plot for the indica panel with GBS data. P-values by chromosome for root cone angle. (JPEG 98 kb) [file 12284_2017_184_MOESM7_ESM.jpg]

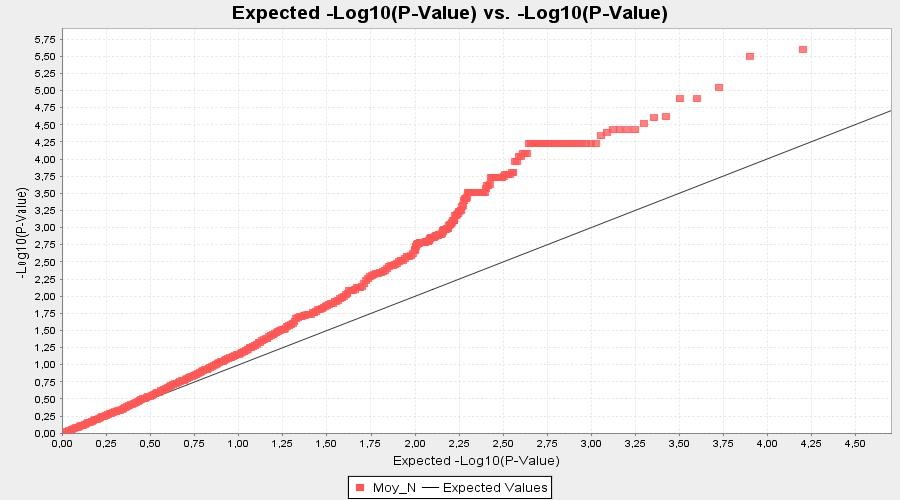

Supplement: Supplementary file 8 — Q-q plot for root cone angle in the japonica panel with GBS data. (JPEG 73 kb) [file 12284_2017_184_MOESM8_ESM.jpg]

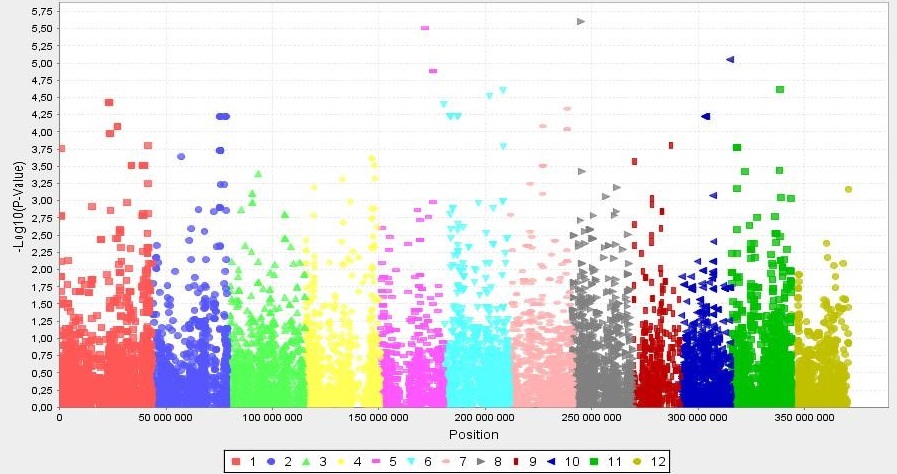

Supplement: Supplementary file 9 — Manhattan plot for the japonica panel with GBS data. P-values by chromosome for root cone angle. (JPEG 142 kb) [file 12284_2017_184_MOESM9_ESM.jpg]

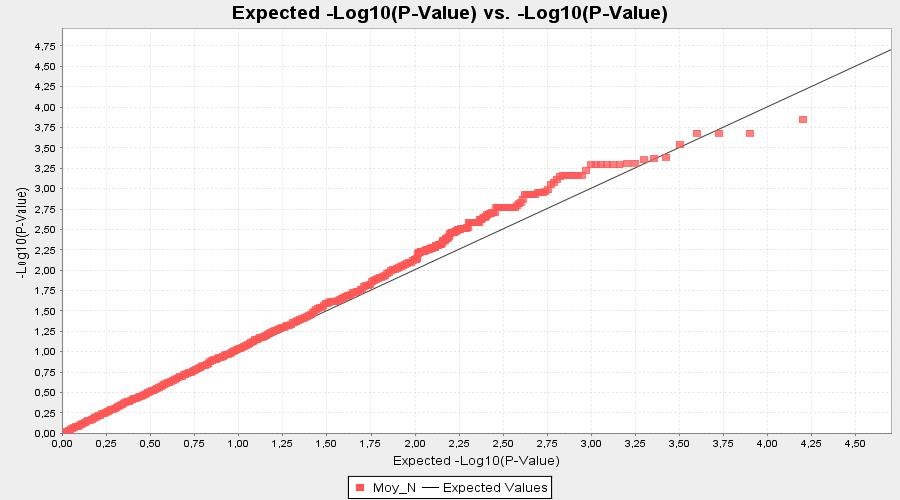

Supplement: Supplementary file 10 — Q-q plot for root cone angle for the japonica panel, 10 extreme lines excluded, with GBS data. (JPEG 67 kb) [file 12284_2017_184_MOESM10_ESM.jpg]

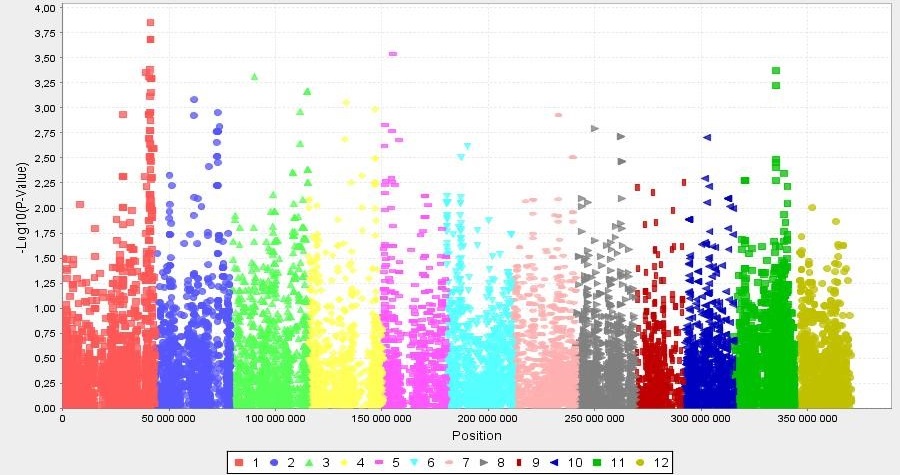

Supplement: Supplementary file 11 — Manhattan plot for the japonica panel, 10 extreme lines excluded, with GBS data. P-values by chromosome for root cone angle. (JPEG 131 kb) [file 12284_2017_184_MOESM11_ESM.jpg]
